# Hematobin is a novel immunomodulatory protein from the saliva of the horn fly *Haematobia irritans* that inhibits the inflammatory response in murine macrophages

**DOI:** 10.1186/s13071-018-3017-z

**Published:** 2018-07-27

**Authors:** Martin Breijo, Eliane Esteves, Bruna Bizzarro, Priscila G. Lara, Josiane B. Assis, Sergio Rocha, Lucía Pastro, Cecilia Fernández, Ana Meikle, Anderson Sá-Nunes

**Affiliations:** 1 Unidad de Reactivos y Biomodelos de Experimentación, Facultad de Medicina, Universidad de la República, Gral. Flores, 2125 Montevideo, Uruguay; 2 Department of Immunology, Laboratory of Experimental Immunology, Institute of Biomedical Sciences, University of Sao Paulo, Sao Paulo, SP 05508-000 Brazil; 3 Laboratorio de Interacciones Moleculares, Facultad de Ciencias, Universidad de la República, Iguá, 4225 Montevideo, Uruguay; 4 Cátedra de Inmunología, Facultad de Química, Universidad de la República, Av. Alfredo Navarro, 3051 Montevideo, Uruguay; 5 Laboratorio de Técnicas Nucleares, Facultad de Veterinaria, Universidad de la República, Lasplaces, 1550 Montevideo, Uruguay; 6 National Institute of Science and Technology in Molecular Entomology, National Council of Scientific and Technological Development (INCT-EM/CNPq), Rio de Janeiro, RJ Brazil

**Keywords:** *Haematobia irritans*, Saliva, Hematobin, Macrophages, Inflammation, Immunomodulatory activity

## Abstract

**Background:**

The horn fly *Haematobia irritans* is a blood-sucking ectoparasite responsible for substantial economic loss of livestock. Like other hematophagous arthropods species, the successful blood-feeding of *H. irritans* is highly dependent on the modulation of the host’s hemostasis and immune system. Here, we evaluated the biological activity of hematobin (HTB), a protein recently identified in the *H. irritans* saliva, on macrophage biology. The goal was to understand the putative interactions between the components of *H. irritans* saliva and the early host immune responses.

**Results:**

Thioglycolate-elicited peritoneal macrophages from BALB/c mice were stimulated by lipopolysaccharide (LPS) plus interferon-γ (IFN-γ) in the presence or absence of recombinant HTB. The presence of the salivary protein in the cultures inhibited nitric oxide production and decreased the inducible nitric oxide synthase (iNOS) expression induced by LPS plus IFN-γ. The tumor necrosis factor-α (TNF-α) and interleukin-12p40 (IL-12p40) levels were also reduced in the macrophages pre-incubated with HTB; these findings correlated to the decreased NF-κB expression. The biological activities described here were not associated with changes in annexin V binding to macrophages suggesting that HTB does not induce cell death. In addition, the activity of HTB seems to be specific to macrophages because no changes were observed in lymphocyte proliferation or cytokine production.

**Conclusions:**

We describe here the first bioactive salivary protein of *H. irritans.* We characterized its ability to modulate macrophage inflammatory response, and the results can help explain how horn flies modulate the host immune system to feed on blood.

**Electronic supplementary material:**

The online version of this article (10.1186/s13071-018-3017-z) contains supplementary material, which is available to authorized users.

## Background

Horn flies (*Haematobia irritans*) are blood-feeding parasites that affect the health and well-being of pasture cattle. The bites can lead to reduced weight gain, less milk production [1] and skin lesions [2]. The economic losses caused by *H. irritans* vary among regions because environmental conditions significantly affect the growth of horn fly populations. In North America, the economic impact of horn flies on livestock is estimated at 1 billion dollars per year [3]. Like nearly all hematophagous arthropod species, the success of blood-feeding in *H. irritans* is highly dependent on its complex salivary mixture delivered at the bite site. It is well known that saliva from hematophagous arthropods facilitates the availability of blood through control of the host hemostasis and immune system [4]. Although several bioactive molecules have been reported in the saliva of mosquitoes, sand flies, horse flies and ticks, knowledge on the functional salivary proteome of *H. irritans* remains limited [5–9].

Many details about the antihemostatic activities of *H. irritans* salivary preparations were described 20 years ago [10]. Thrombostasin was the first protein identified and characterized in horn fly saliva as an anticlotting molecule [11]. Recently, two additional proteins [Irritans 5 (IT5) and Hematobin (HTB)] have been annotated in a preliminary cDNA library preparation of *H. irritans* salivary gland (M. Breijo, personal communication). IT5 belongs to the antigen 5 (Ag5) family, whose members were reported to be allergens of the sting venom of hornets, wasps and fire ants [12]; it has 86% similarity with the Ag5 protein found in the transcriptome of the stable fly, *Stomoxys calcitrans*. Ag5 is likely an immunoglobulin-binding protein [13], and thus counteracts the host’s immunoglobulins present in the blood [7].

HTB has 43% similarity with a putative 15.6 kDa secreted salivary gland protein of *S. calcitrans*, which is a member of the Hyp 16 family with unknown function that is distantly related to mosquito proteins [14]. The functions of HTB and IT5 in the saliva of *H. irritans* are still unknown, but they may play a role in vector-host interactions, particularly on the skin environment at the horn fly bite site.

Skin is the primary interface between the vertebrate host and the external environment. It provides an active barrier against microorganisms and ectoparasites. Many resident immune cells are present in the skin, particularly in the dermis. These include dendritic cells, conventional T cells, innate lymphoid cells, mast cells, and macrophages [15, 16]. Of those, macrophages are pleiotropic cells presenting both pro-inflammatory and anti-inflammatory activities depending on the stimuli. They are the predominant cell population at the site [16]. Macrophages play a crucial role in the response to inflammation and tissue damage while promoting homeostasis to assure proper reestablishment of normal conditions.

Due to their strategic location, skin macrophages might be one of the first sensors to detect and respond to *H. irritans* mouthparts and salivary components. Indeed, breaking the skin barrier during blood-feeding triggers inflammatory responses, and macrophages recognize the presence of the causative agents through pattern recognition receptors that activate several intracellular pathways. This induces expression of transcription factors and enzymatic cascades that lead to the production of inflammatory molecules such as cytokines, free radicals, and lipid mediators [17]. However, it is not known whether HTB or other horn fly salivary proteins affect macrophage biology. Here, we explored the potential role of a recombinant HTB on biological parameters of classically activated murine macrophages.

## Methods

### Mice

Female BALB/c mice, 6–12 weeks-old, were bred and maintained at the Isogenic Breeding Unit (Department of Immunology, Institute of Biomedical Sciences, University of Sao Paulo) under specific pathogen-free conditions.

### Identification, production, and purification of HTB

HTB (GenBank: AJY26992.1) was identified in a cDNA library prepared from *H. irritans* salivary glands. The production of recombinant HTB was performed as described [18]. Briefly, the sequence coding for the predicted mature HTB was amplified by PCR from a recombinant plasmid carrying the full-length cDNA derived from an oligo-capped full-length enriched library prepared from the *H. irritans* salivary gland mRNA. The fragment was amplified with primers carrying sites for KpnI and SalI and cloned into a KpnI/SalI-cut pQE-30 vector (Qiagen, Hilden, Germany). The plasmid was used to transform *Escherichia coli* M15 cells, and protein expression was induced using 0.1 mM isopropyl-D-thiogalactopyranoside for 2 h at 37 °C. The N-terminal 6× histidine-tagged HTB was purified using a nickel-nitrilotriacetic acid agarose column (Qiagen) under denaturing conditions following the manufacturer’s instructions. Antigenic similarity between native and recombinant proteins was confirmed by Western blot using anti-HTB polyclonal rabbit sera blotted against *H. irritans* salivary gland extract (Additional file 1: Figure S1). The alignment was performed by MUSCLE [19] and graphically edited using BioEdit software [20].

### Peritoneal macrophage cultures

Mice were injected intraperitoneally with 1 ml of 4% sterile thioglycollate solution (BD-Difco, Franklin Lakes, NJ, USA). Four days later, the mice were euthanized, and the peritoneal lavage was collected after injection of 5 ml cold RPMI 1640 medium (Gibco^TM^, Grand Island, NY, USA). After centrifugation, cells were resuspended in RPMI 1640 medium, counted, diluted at 2 × 10^6^ peritoneal cells/ml, and distributed in 24- or 96-well flat bottom plates (depending on the assay) at a volume of 1 ml/well or 100 μl/well, respectively. After 2 h of incubation in a humidified atmosphere of 5% CO_2_ at 37 °C, the non-adherent cells were removed by 3 washes with warm sterile phosphate-buffered saline (PBS). The adherent cells were considered to be macrophages and were pre-incubated overnight with complete medium only (RPMI 1640 supplemented with 10% of heat inactivated fetal bovine serum (FBS), 2 mM L-glutamine, 25 mM HEPES, 55 μM 2-mercaptoethanol, 100 U/ml penicillin, and 100 μg/ml streptomycin; all from Gibco^TM^, Grand Island, NY, USA) or with different concentrations of HTB (125, 250, 500 and 1000 nM) diluted in complete medium. After that, the cells were stimulated with murine recombinant interferon-γ [IFN-γ (10 ng/ml; Sigma-Aldrich, St. Louis, MO, USA)] plus ultrapure lipopolysaccharide [LPS (10 ng/ml; InvivoGen, San Diego, CA, USA)] and cultured for different times according to the assay.

### Nitric oxide (NO), cytokines, and prostaglandin E_2_ (PGE_2_) determinations

Macrophage cultures were prepared and stimulated as described above. Cell-free supernatants of these macrophage cultures were then collected for measurements of NO (48 h), cytokines (6 h and 24 h), and PGE_2_ (24 h). The Griess reaction determined nitrite (NO_2_^-^), a stable product of NO oxidation, as previously described [21, 22]. An enzyme-linked immunosorbent assay (ELISA) was used to determine tumor necrosis factor-α (TNF-α) and interleukin-12p40 (IL-12p40) levels (OptEIA ELISA Sets - BD Biosciences, San Diego, CA, USA). The PGE_2_ concentrations were measured according to the manufacturer’s instructions (PGE_2_ ELISA Kit; Cayman Chemical, Ann Arbor, MI, USA).

### Western blot analysis

Macrophage cultures were prepared and stimulated as described above. After 30 min [for nuclear factor-κB (NF-κB)], 6 h [for cyclooxygenase-2 (COX-2)], or 24 h [for inducible nitric oxide synthase (iNOS)], the supernatant was removed and the adherent cells were lysed with RIPA buffer (150 mM NaCl, 1% NP40, 0.1% SDS, 50 mM Tris, pH 8.0) supplemented with phosphatase inhibitors (100 mM sodium fluoride and 100 mM sodium orthovanadate) and 1% protease inhibitor (Sigma-Aldrich). The cell lysates were then centrifuged at 14,000× *g* for 10 min, and the protein concentration was measured by the BCA Protein Assay Kit (Thermo Fisher Scientific, Waltham, MA, USA) according to the manufacturer’s instructions. The same amount of protein for each sample was mixed with the Bolt™ Sample Reducing Agent and Bolt™ LDS Sample Buffer (Invitrogen, Carlsbad, CA, USA) and boiled at 70 °C for 10 min. The samples were then separated on Bolt™ Bis-Tris Plus Gels 4–12%, and the proteins were transferred to nitrocellulose membranes using iBlot® Dry Blotting System (Invitrogen).

The membranes were blocked for 2 h with 10% FBS in Tris buffer at pH 7.5 containing 1% of Tween-20 (TBST). This was washed with TBST three times (5 min per wash) and incubated overnight at 4 °C with the following monoclonal antibodies: anti-iNOS (1:10,000; Cell Signaling Technology Inc., Danvers, MA, USA), anti-COX-2 (1:1000; Cayman Chemical, Ann Arbor, MI, USA), and anti-phospho NF-κB p65 (1:1000; Cell Signaling Technology Inc., Danvers, MA, USA). After further washing, the membranes were incubated for 1 h at room temperature with anti-rabbit secondary antibodies (1:3000) conjugated with horseradish peroxidase for detection (Cell Signaling Technology Inc.). Immunoreactive bands were stained using the Novex® Chemiluminescent Substrate Reagent Kit (Invitrogen) and visualized in a photodocumentation system G:BOX (Syngene, Cambridge, UK). The membranes were then washed and incubated for 1 h with anti-β-actin conjugated with horseradish peroxidase (1:10,000; Sigma-Aldrich). The immunoreactive bands were again stained and visualized as described. The density of the bands was analyzed using AlphaDigiDoc™ System 1000 software version 3.2 Beta (Alpha Innotech, San Leandro, CA, USA).

### Flow cytometry

Macrophage cultures were prepared as described above. After 24 h, the cells were collected, washed, and stained with fluorochrome-conjugated anti-F4/80 (a macrophage marker antibody) and anti-CD40 (an activation marker). Cells were incubated for 30 min in the dark, washed, re-suspended in PBS/FBS 1%, and analyzed by flow cytometry. Annexin V staining was used to evaluate cell death. Cells were washed and re-suspended with annexin-binding buffer (0.1 M HEPES, 1.4 M NaCl, and 25 mM CaCl_2_) containing annexin V properly diluted. This was then incubated in the dark for 10 min at room temperature. The percentage of annexin V^+^ cells was evaluated by flow cytometry. Spleen cell preparations were also incubated with salivary gland extract (SGE) of *Aedes aegypti* and ran in parallel as a positive control for the cell death assay [23]. Cells were acquired in a FACSCanto II (BD Biosciences San Diego, CA, USA), and the results were analyzed using the FlowJo software (Tree Star Inc., Ashland, OR, USA).

### Lymphocyte proliferation and cytokine production

Spleens were aseptically removed from mice, and a suspension containing 10^6^ cells/ml was prepared in complete medium. This was then distributed in 24-well flat bottom plates at 1 ml aliquots/well, and pre-incubated overnight with complete medium only or with different concentrations of HTB (125, 250, 500 and 1000 nM). The cultures were stimulated with concanavalin A [(Con A) 1 μg/ml final concentration - Sigma-Aldrich], a polyclonal mitogen of T cells, and incubated in a humidified atmosphere of 5% CO_2_ at 37 °C. After 48 h of incubation, 25 μl of a 0.01% resazurin solution was prepared in complete medium and added to each well followed by 24 h of additional incubation. The proliferation was calculated as the subtraction of the absorbance at 570 and 600 nm for each well, as previously described [23, 24].

Some cultures were prepared in parallel using a suspension containing 5 × 10^6^ cells/ml. The cells were distributed in 96-well flat bottom plates at 100 μl aliquots/well and stimulated similarly. After 72 h, cell-free supernatants were removed to determine IFN-γ, IL-4 and IL-5 concentrations by OptEIA ELISA Set (BD Biosciences) according to the manufacturer’s instructions.

### Statistical analysis

The statistical analyses of differences between means of experimental groups were performed using analysis of variance (ANOVA) followed by Tukey’s *post-hoc* test. A value of *P* < 0.05 was considered significant. Data are shown as the mean ± standard error of the mean (SEM).

## Results

### HTB sequence and alignment

**Fig. 1:**
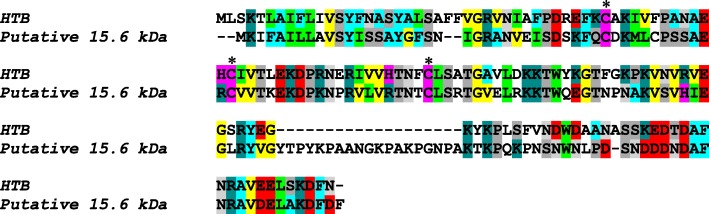
Alignment of HTB and the putative 15.6 kDa salivary protein of *S. calcitrans*. The amino acid sequence of *H. irritans* HTB (accession number AJY26992.1) was compared to a *S. calcitrans* putative 15.6 kDa salivary gland protein (accession number NP_001298181.1). The alignment was performed using MUSCLE method and graphically edited using BioEdit software. Asterisks highlight the conserved cysteine residues. The threshold for shading colors of amino acid similarity was 40%

The HTB gene was annotated as a sequence of 598 nucleotides coding for a protein of 143 amino acids length presenting a signal peptide of 20 amino acids based on a cDNA library of *H. irritans* salivary glands. The mature protein has an estimated molecular weight of 13.89 kDa and an isoeletric point of 8.47. BLAST searches revealed that the unique sequence of HTB (GenBank: AJY26992.1) has approximately 43% similarity to a salivary protein of *S. calcitrans* of unknown function and annotated as a putative 15.6 kDa secreted salivary gland protein (GenBank: NP_001298181.1) (Fig. 1).

### HTB inhibits NO production and iNOS expression by activated macrophages

**Fig. 2:**
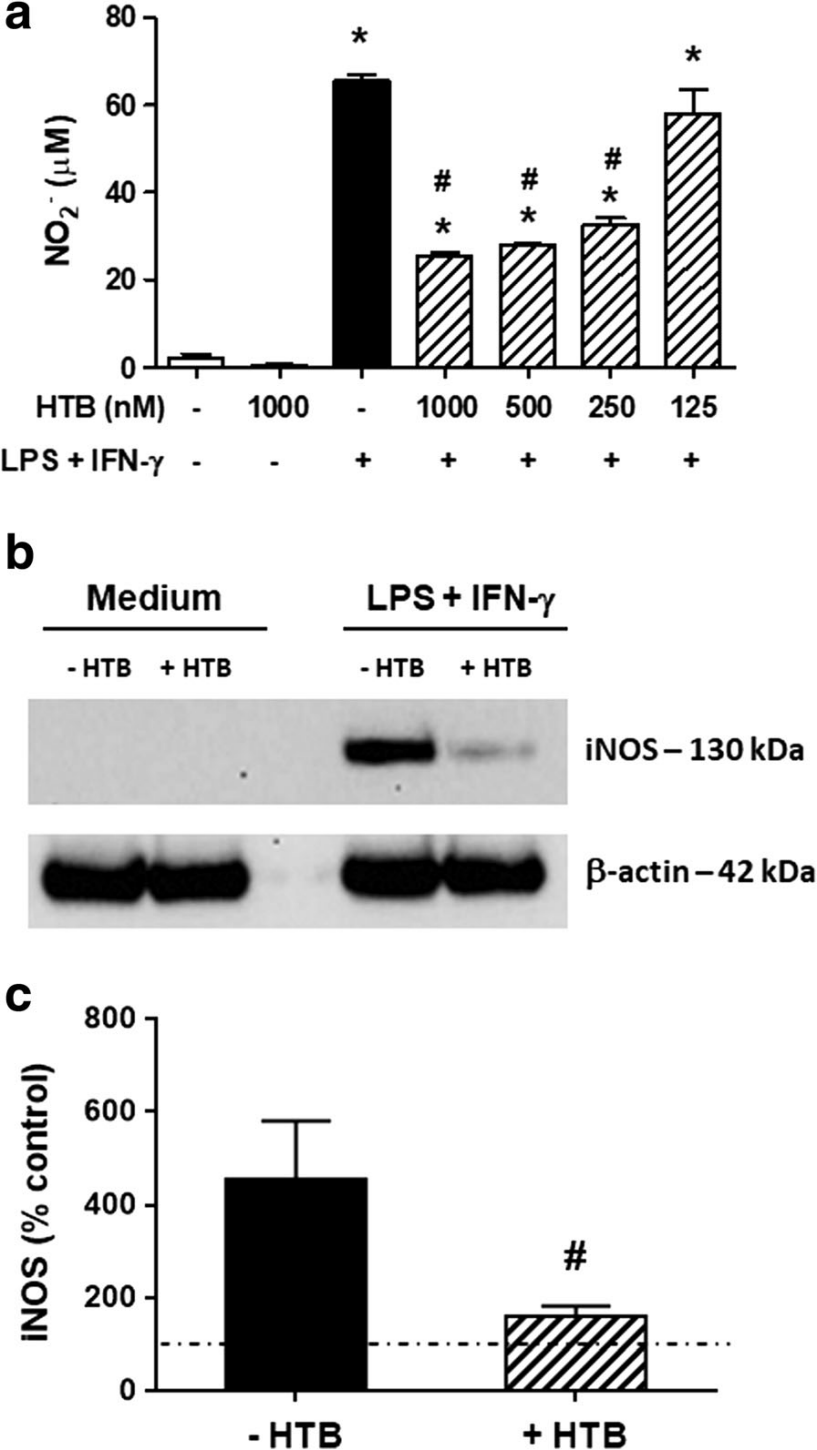
HTB decreases NO production and iNOS expression by activated macrophages. Macrophages were pre-incubated overnight with HTB (125 to 1000 nM) and stimulated with LPS plus IFN-γ (10 ng/ml of each). Nitric oxide production was measured after 48 h in cell-free supernatants by Griess reaction (**a**). The iNOS expression was evaluated after 24 h in cell lysates by Western blot (**b**). The expression of iNOS and β-actin were determined by densitometry analysis of the bands. Results are presented as a percentage of the control, which corresponds to unstimulated cells (**c**). Data represent mean ± SEM. *P < 0.05 *versus* control and ^#^*P* < 0.05 *versus* LPS/IFN-γ-activated cells in the absence of HTB

We first evaluated the effect of HTB on the NO production and iNOS expression by macrophages activated with IFN-γ plus LPS. Macrophages maintained in medium or in the presence of HTB produced almost undetectable levels of NO, while activation with IFN-γ plus LPS induced a significant production of this mediator (*F*_(6, 21)_ = 124.8, *P* < 0.0001). The incubation of macrophages with increasing concentrations of HTB before IFN-γ/LPS activation prevented NO production in a concentration-dependent manner (Fig. 2a). This reached a maximum of 60% inhibition at 1000 nM (*F*_(6, 21)_ = 124.8, *P* < 0.0001). Accordingly, no expression of iNOS was detected in lysates from macrophages maintained in medium or HTB only. On the other hand, activated macrophages expressed iNOS as expected, while pre-incubation with HTB decreased the enzyme expression induced by activation (Fig. 2b). The densitometry of the bands from four independent experiments (Fig. 2c) confirms the inhibition of iNOS expression in the presence of HTB (*F*_(3, 12)_ = 7.15, *P* = 0.0274).

### HTB inhibits the production inflammatory cytokines and NF-κB expression in activated macrophages

**Fig. 3:**
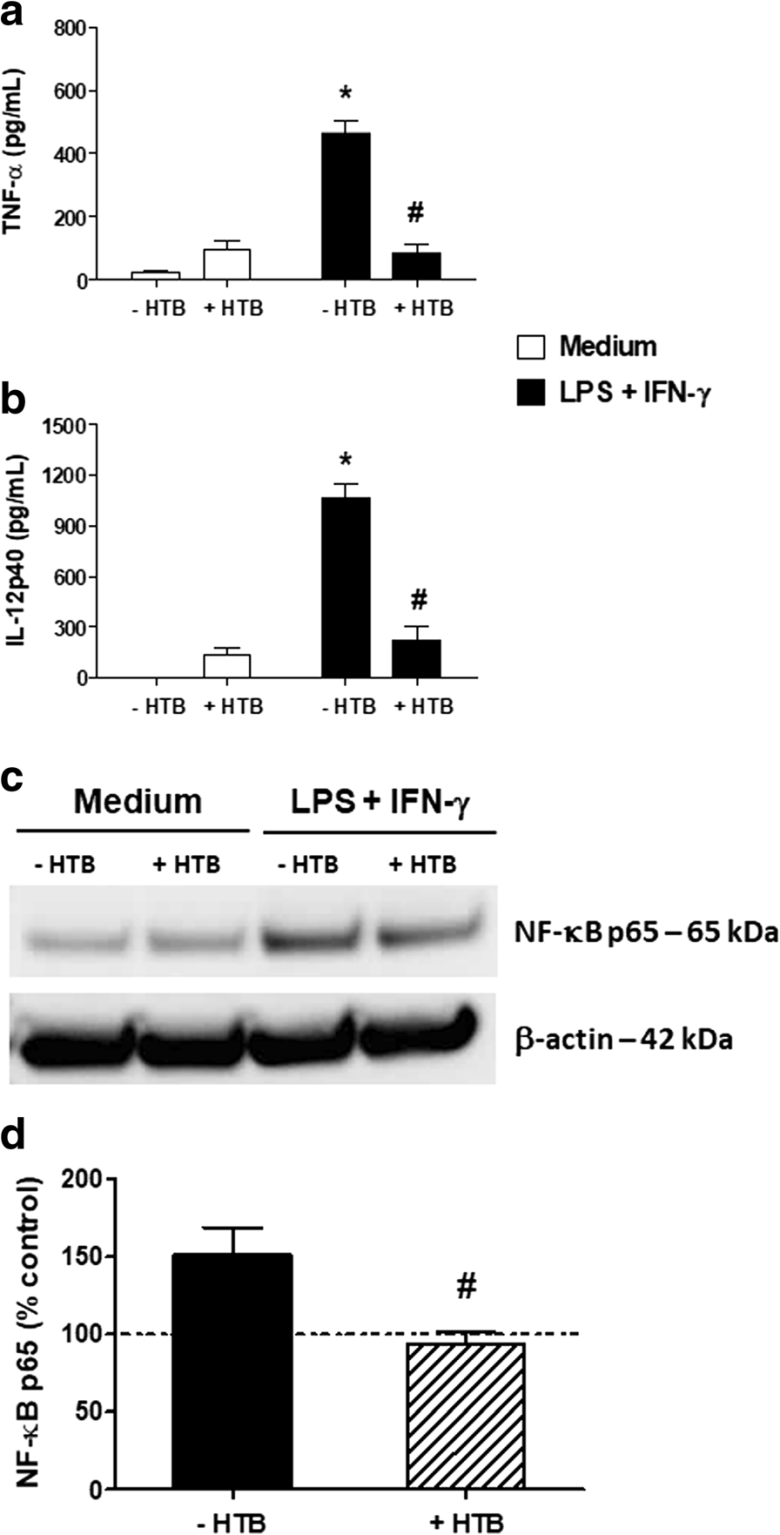
HTB inhibits inflammatory cytokine production and NF-κB phosphorylation by activated macrophages. Macrophages were pre-incubated overnight with HTB (1000 nM) and stimulated with LPS plus IFN-γ (10 ng/ml of each, final concentration). The production of TNF-α (**a**) and IL-12p40 (**b**) were detected after 6 h and 24 h, respectively, in cell-free supernatants by ELISA. The phosphorylation of NF-κB was evaluated after 30 min in cell lysates by Western blot (**c**). The expression of phosphorilated NF-κB and β-actin were determined by densitometry analysis of the bands. The results are presented as a percentage of the control, which corresponds to unstimulated cells (**d**). The data represent the mean ± SEM. *P < 0.05 *versus* control and ^#^*P* < 0.05 *versus* LPS/IFN-γ-activated cells

We next evaluated whether HTB could affect the production of inflammatory cytokines produced as a consequence of macrophage activation. Macrophages maintained in medium or HTB produced low basal levels of TNF-α (Fig. 3a) or IL-12p40 (Fig. 3b). Activation with IFN-γ plus LPS induced significant production of both cytokines (*F*_(3, 8)_ = 46.97, *P* < 0.0001 for TNF-α; *F*_(3, 8)_ = 61.29, *P* < 0.0001 for IL-12p40), and the pre-incubation with HTB strongly inhibited their production (*F*_(3, 8)_ = 46.97, *P* < 0.0001 for TNF- α; *F*_(3, 8)_ = 61.29, *P* < 0.0001 for IL-12p40). Under these conditions, very low levels of IL-10 were detected, and the presence of HTB did not significantly affect this production (data not shown). The expression of NF-κB, a key transcription factor involved in the production of a number of inflammatory cytokines, was evaluated in cell lysates. Basal expression of NF-κB was detected in lysates from macrophages maintained in medium or HTB only. Activated macrophages had increased NF-κB expression, while pre-incubation with HTB decreased the transcription factor expression in these cells (Fig. 3c). The densitometry of the bands (Fig. 3d) from three independent experiments confirmed this difference (*F*_(3, 8)_ = 6.978, *P* = 0.0135).

### HTB did not affect PGE_2_ production and COX-2 expression by activated macrophages

**Fig. 4:**
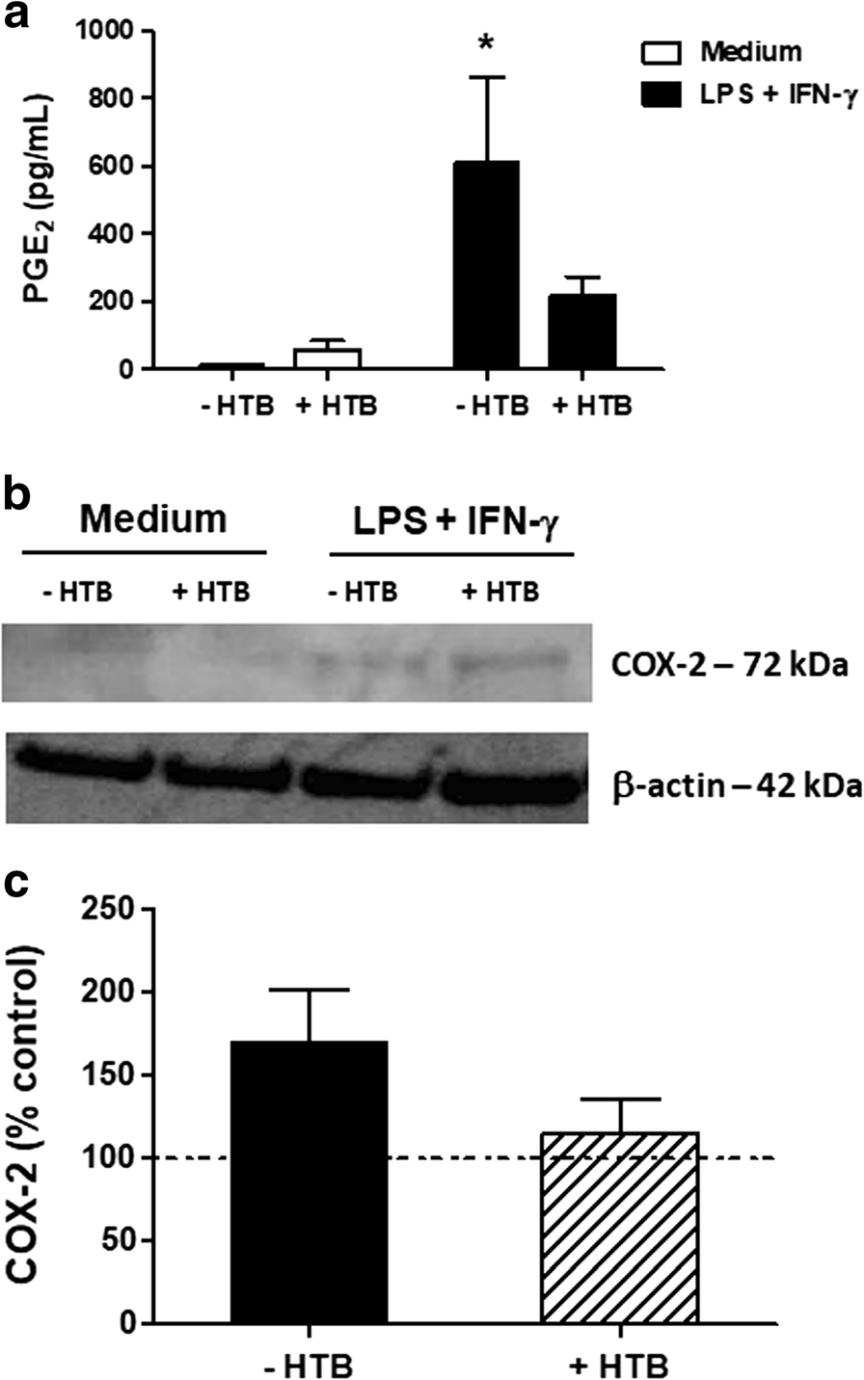
HTB partially affects PGE_2_ production and COX-2 expression by activated macrophages. Macrophages were pre-incubated overnight with HTB (1000 nM) and stimulated with LPS plus IFN-γ (10 ng/ml of each). The PGE_2_ production was measured after 24 h in cell-free supernantants by ELISA (**a**). The expression of COX-2 was evaluated after 6 h in cell lysates by Western blot (**b**). The expression of COX-2 and β-actin was determined by densitometry analysis of the bands. The results are presented as a percentage of the control, which corresponds to unstimulated cells (**c**). The data represent the mean ± SEM. *P < 0.05 *versus* control

Macrophage activation is also associated with the production and release of lipid mediators, such as PGE_2_. Thus, we evaluated if HTB would also affect PGE_2_ production as well as the expression of COX-2, the enzyme involved in PGE_2_ synthesis. As observed for NO and inflammatory cytokines, macrophages maintained in medium or HTB released low amounts of PGE_2_, and activation with IFN-γ and LPS induced significant production of this lipid mediator (*F*_(3, 32)_ = 4.392, *P* = 0.0133). Although pre-incubation with HTB inhibited the PGE_2_ production by more than 60%, this difference was not statistically significant due to the individual variation of each animal (Fig. 4a). A similar pattern was observed for COX-2 expression evaluated in macrophage lysates. Basal expression was observed for macrophages maintained in medium or HTB only, while IFN-γ plus LPS activation increased its expression (Fig. 4b). In the presence of HTB, activated macrophages did not significantly reduce the expression of COX-2, as seen in the densitometry of the bands representative of five experiments (Fig. 4c).

### HTB negatively modulates CD40 expression in activated macrophages

**Fig. 5:**
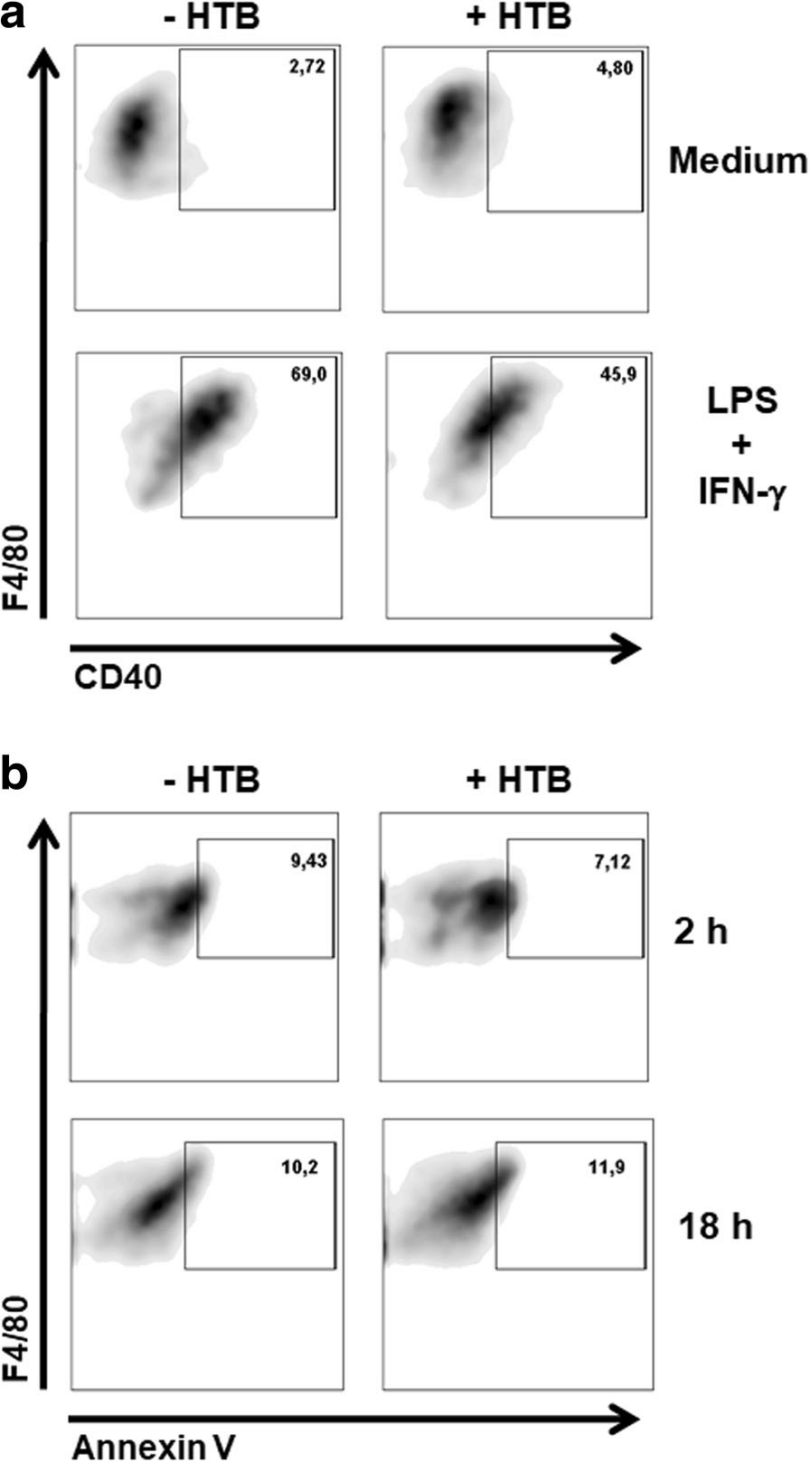
HTB downmodulates CD40 expression by activated macrophages and does not induce cell death. Macrophages were pre-incubated overnight with HTB (1000 nM) and stimulated with LPS plus IFN-γ (10 ng/ml of each). Flow cytometry was employed to evaluate the CD40^+^ events in F4/80^+^ gated cells after 24 h (**a**); and annexin V^+^ events in F4/80^+^ gated cells after 2 h and 18 h (**b**). The data are expressed as the percentage of positive events for each marker

The biological activity of HTB was also evaluated on the expression of CD40, a cell surface marker associated with macrophage activation. Macrophages incubated with medium only express a discrete amount of CD40 on its surface, and the presence of HTB does not interfere with this expression. Stimulation with LPS and IFN-γ increases the expression of CD40 as a sign of macrophage activation while HTB partially impaired its expression suggesting that HTB has a negative effect on macrophage activation (Fig. 5a).

### HTB does not induce macrophage cell death

We next evaluated whether the anti-inflammatory effects of HTB are *via* its toxicity. The macrophage death was determined in the presence of HTB, and annexin V staining showed that the incubation of macrophages with HTB did not induce phosphatidylserine on the outer membrane after 2 h or 18 h (Fig. 5b). As an internal control, a similar assay was performed with lymphocytes and *A. aegypti* SGE. This showed extensive annexin V staining of these cells under the same conditions (Additional file 2: Figure S2).

### HTB biological activities are selective to macrophages

**Fig. 6:**
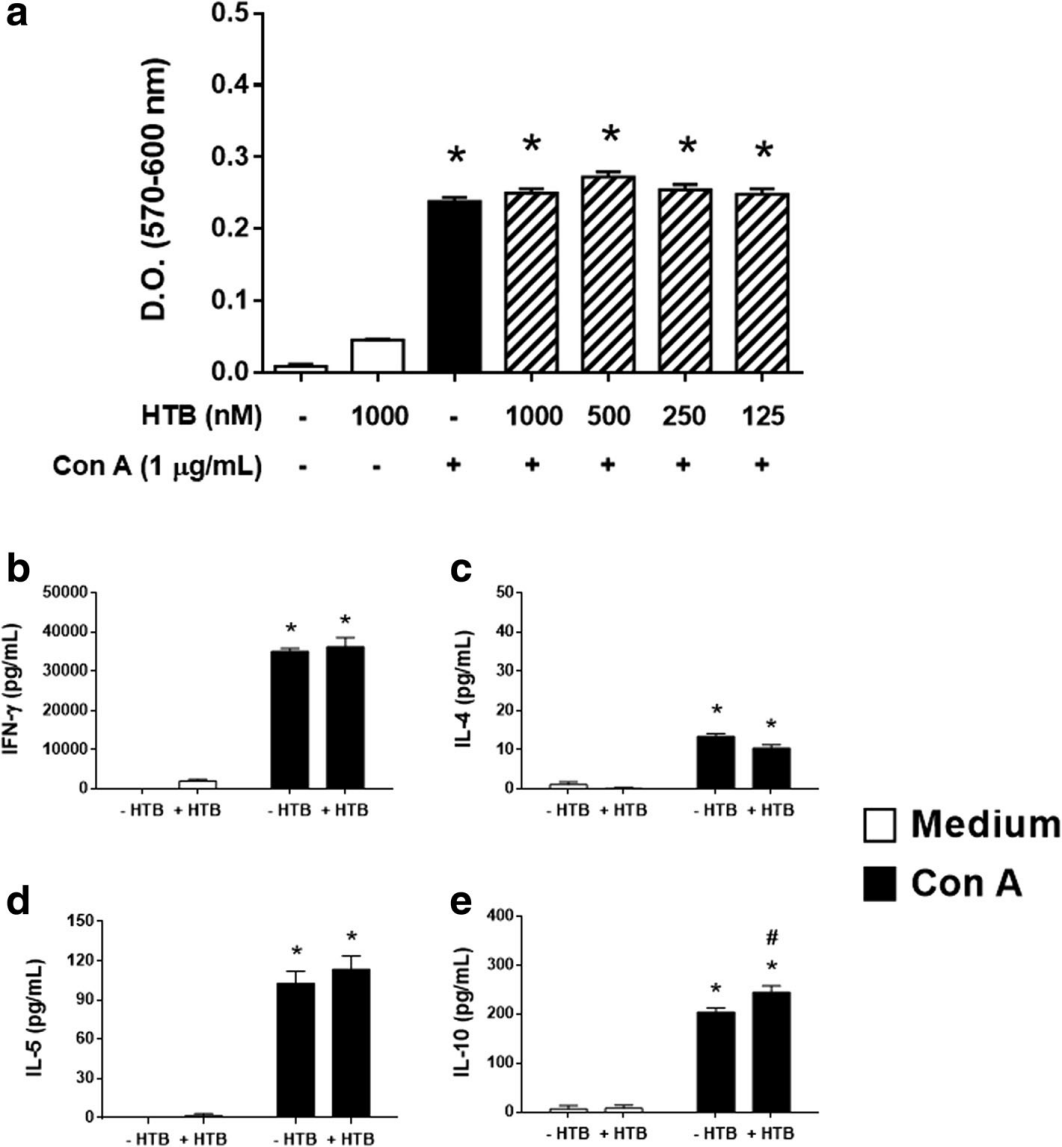
HTB does not affect lymphocyte proliferation or cytokine production. Lymphocytes were pre-incubated overnight with HTB (125 to 1000 nM) and stimulated with Con A (0.5 μg/ml, final concentration). After 72 h, the proliferation was measured by a colorimetric assay (**a**) and the levels of IFN-γ (**b**), IL-4 (**c**), IL-5 (**d**) and IL-10 (**e**) were measured in cell-free supernatants by ELISA. The data represent the mean ± SEM. *P < 0.05 *versus* control and ^#^*P* < 0.05 *versus* LPS/IFN-γ-activated cells

To evaluate the selectivity of HTB effects, we evaluated lymphocyte proliferation and cytokine production in the presence of the protein. Figure 6a shows that Con A-induced lymphocyte proliferation did not change by pre-incubation with any of the HTB concentrations tested. Likewise, the production of IFN-γ (Fig. 6b), IL-4 (Fig. 6c), and IL-5 (Fig. 6d) induced by Con A was not changed in the presence of HTB. However, in Con A-stimulated groups, IL-10 production was slightly increased (*F*_(3, 12)_ = 190.1, *P* = 0.0374) in the presence of HTB (Fig. 6e).

## Discussion

There is little information available on the molecules secreted in *H. irritans* saliva. Thrombostasin, a salivary thrombin inhibitor, is the only protein identified and characterized to date [11]. Here, we used an *in vitro* model that mimics early interactions between microbial products and innate immune responses. This revealed that HTB, a novel horn fly salivary protein, can downmodulate the production of inflammatory mediators by macrophages. The presence of HTB in macrophage cultures significantly reduced the NO production induced by LPS plus IFN-γ activation. NO is constitutively produced by NO synthases in neurons and endothelial cells; however, iNOS is expressed in macrophages and accounts for host resistance to a number of pathogens in the presence of inflammatory stimuli such as LPS and cytokines [25, 26]. Here, reduced iNOS expression was also seen in activated macrophages by the addition of HTB to the cultures. Similar downregulation of NO production and/or iNOS expression by macrophages under various stimuli have been reported in the presence of saliva and salivary components from ticks [27–30], triatomines [31], sandflies [32–37], mosquitoes [38, 39] and horseflies [8].

The inflammatory effect of TNF-α is mainly related with the induction of leukocyte adhesion molecules in the vascular endothelium and the recruitment of cells to the injured site [40]. IL-12p40 is a subunit shared by IL-12 (when linked to the IL-12p35 subunit) and IL-23 (when linked to the IL-23p19 subunit); they are both members of the IL-12 cytokine family [41] and are produced mostly by dendritic cells and macrophages. IL-12 was originally identified as a factor capable of stimulating natural killer cells to produce IFN-γ [42]. Later, it was shown to be the major cytokine involved in polarization of CD4^+^ T cells to the Th1 profile [43–47]. On the other hand, IL-23 is associated with the development of IL-17-producing CD4^+^ T cells (Th17 profile) [48, 49]. In addition, the decreased production of TNF-α and IL-12p40 was associated with downmodulation of NF-κB, one major transcription factor involved in the production of many inflammatory cytokines. Like *H. irritans*, other hematophagous arthropods have components in their saliva that can prevent inflammatory cytokine secretion and/or NF-κB expression by activated macrophages [27, 30, 50–53], and some molecules associated with this immunomodulatory activity have been revealed.

For example, a potent vasodilatory peptide from sand fly saliva, maxadilan, inhibits LPS-induced production of NO, TNF-α, and IL-12 by macrophages [37]. The tick *Ixodes ricinus* has a salivary serpin named Iris (*Ixodes ricinus* immunosuppressor) that inhibits the production of pro-inflammatory cytokines by LPS-stimulated peripheral blood mononuclear cells [54–56]. The protein Cecropin-TY1, previously identified in the horsefly *Tabanus yao* salivary gland as an antimicrobial peptide [57], has potent anti-inflammatory activity on the production of NO and cytokines. It inhibits the activation of mitogen-activated protein kinases (MAPKs) and NF-κB signals in macrophages [8]. Therefore, the inhibition of cytokines and a transcription factor related to their production by HTB might affect the pro-inflammatory milieu provided by activated macrophages during an early inflammatory response. Consequently, the diminished migration of immune cells and the impaired differentiation of Th1/Th17 subpopulations potentially caused by HTB likely benefits horn fly feeding.

PGE_2_ is a lipid mediator from the eicosanoid family involved in a number of physiological, inflammatory and regulatory functions. Under inflammatory conditions (e.g. LPS stimulation or pathogen infection), macrophages and other immune cells express phospholipase A_2_ that releases arachidonic acid from membrane phospholipids. Subsequently, arachidonic acid is oxidized by COX-2 and modified into PGE_2_ by microsomal PGE synthase-1 [58]. Given the importance of the PGE_2_ in inflammation and immunity to pathogens, we analyzed whether HTB could modulate macrophage secretion of PGE_2_ and/or COX-2 biosynthesis. The presence of HTB in the culture did not change PGE_2_ secretion by resting or activated macrophages. A similar phenotype was achieved for COX-2 expression in macrophage lysates. Thus, the PGE_2_/COX-2 axis is not consistently modulated by HTB.

CD40 is a surface membrane molecule expressed by antigen-presenting cells and other cell types such as mast cells, fibroblasts and endothelial cells. The interactions between CD40 in macrophages and its ligand (CD40L, also known as CD154) in T cells provide bidirectional stimulatory signals to these cells [59]. CD40 engagement induces robust activation of pro-inflammatory cytokine synthesis in macrophages, including TNF-α, IL-12, IL-1α, IL-1β and chemokines as well as NO production, upregulation of MHC class II expression, and co-stimulatory molecules [60]. All of these factors can amplify inflammatory responses of T cells. We observed that CD40 expression was highly increased after stimulation with LPS and IFN-γ, but the presence of HTB in the cultures partially prevented its expression in macrophages, this confirms the anti-inflammatory role of HTB in these cells.

Salivary preparations from some hematophagous arthropods have recently been shown to induce programmed death to a number of cell types. The SGE of the sand fly *Lutzomyia longipalpis* induces apoptosis in neutrophils [61] while SGE of the mosquito *A. aegypti* directly induces apoptosis in lymphocytes [23]. The SGE of the mosquito *Armigeres subalbatus* decreased the expression of TNF-α, IFN-β, CXCL10 and iNOS as well as NO production by macrophages. This downmodulation was associated with apoptotic cell death in these cells and in lymphocytes [39]. Thus, to evaluate whether the anti-inflammatory activities described were actually due to cell death, we compared annexin V binding to activated macrophages incubated in the presence or absence of HTB and observed no significant differences in the binding between these two conditions. HTB neither affected lymphocyte metabolism/proliferation nor cytokine production upon polyclonal activation suggesting the absence of toxicity and a selective mechanism of action of the protein.

Overall, our results suggest that at least one component of *H. irritans* saliva can modulate inflammatory responses mediated by classically activated macrophages in a vertebrate host. This is consistent with the fact that *H. irritans* most frequently feeds on cattle, and they offer a more convenient environment in the host skin by controlling inflammatory responses triggered by the fly bite. The skin is a highly complex organ interspersed with a variety of cell types (macrophages, dendritic cells, keratinocytes, mast cells and innate lymphoid cells, among others) and all of them express pattern recognition receptors and produce pro-inflammatory chemokines and cytokines in response to pathogenic stimuli [62]. Further studies are needed to understand in depth the ability of HTB to modulate different cell types in the early response to the injury in the skin.

Few studies compared the effect of arthropod salivary components among species side by side. Regarding ticks, the saliva of *Amblyomma* (*cajennense*) *sculptum* inhibits lymphocyte proliferation of mice and horses [63]. *Ixodes ricinus* saliva has a protein that binds murine and human TNF-α [64], while saliva of *Ixodes scapularis* contains a protein with binding properties against murine and human IL-2 [65]. The salivary gland extract of the mosquito *A. aegypti* kills lymphocytes from mice and guinea-pigs (Anderson Sá-Nunes, personal communication). We cannot assure whether HTB would have the same effect on cattle macrophages. However, considering the similarities of macrophage biology among vertebrate species in terms of functions and intracellular signaling cascades, this is an interesting topic for future consideration.

Finally, our findings lend support to the hypothesis that cattle resistance to horn fly-borne infections could be enhanced by blocking the activities of salivary molecules. HTB is a putative candidate for vaccination. In fact, HTB was recently evaluated as an immunogen in a preliminary field trial, and it reduced *H. irritans* loads by 30% in cattle [66]. The evaluation of different vaccination protocols may improve these findings.

## Conclusions

Here, we reported the first salivary protein of *H. irritans* with immunomodulatory functions. HTB can negatively modulate macrophage-derived inflammatory mediators, its activity is selective to macrophages, and it is not associated with cell death. This work is an important contribution to unveil the functional salivary proteome of *H. irritans* and opens new venues to the evaluation of potential saliva-based vaccines to protect cattle from horn fly infestations.

## Additional files

Additional file 1: **Figure S1.** Evaluation of the cross-reactivity between native and recombinant HTB. Whole salivary gland extract of *H. irritans* was separated on a 12% gradient poly-acrylaminde gel and transferred onto a PVDF membrane. The membranes were blocked with PBS containing 5% of soy milk and were probed with either pre-immune rabbit serum or with serum from rabbits immunized with the recombinant HTB. Then, the membranes were incubated with HRP-conjugated anti-rabbit IgG and bands were detected with 3,3’-Diaminobenzidine. Lane A: pre-immune rabbit serum; Lane B: serum from rabbit immunized with recombinant HTB (immune serum). The immune serum recognized a single band with approximately 15 kDa from the salivary gland extract corresponding to the native protein. MW: molecular weight. (TIF 127 kb)

Additional file 2: **Figure S2.** *Aedes aegypti* SGE induces lymphocyte death *in vitro*. Spleen cells were cultured in the presence of *A. aegypti* SGE and stimulated by Con A (0.5 μg/ml, final concentration) according to [23] as a positive control for the assay presented in Fig. 5. Flow cytometry evaluated annexin V^+^ events in CD3^+^-gated cells after 2 h and 18 h. The data are expressed as the percentage of annexin V^+^ events for each condition. (TIF 115 kb)

## Abbreviations

Ag5: Antigen 5
ANOVA: Analysis of variance
CD: Cluster of differentiation
Con A: Concanavalin A
COX-2: Cyclooxygenase-2
FBS: Fetal bovine serum
HTB: Hematobin
IFN-γ: Interferon-γ
IL: Interleukin
iNOS: Inducible nitric oxide synthase
IT5: Irritans 5
LPS: Lipopolysaccharide
MAPK: Mitogen-activated protein kinase
NF-κB: Nuclear factor kappa-B
NO: Nitric oxide
NOD: Nucleotide-binding oligomerization domain
PBS: Phosphate-buffered saline
PGE_2_: Prostaglandin E_2_
RPMI: Roswell Park Memorial Institute Medium
SDS: Sodium dodecyl sulfate
SEM: Standard error of the mean
SGE: Salivary gland extract
TBST: Saline-buffered with Tris/tween-20
TNF-α: Tumor necrosis factor-α
TS: Thrombostasin

## References

[1] Oyarzun MP, Quiroz A, Birkett MA (2008). Insecticide resistance in the horn fly: alternative control strategies. Med Vet Entomol..

[2] Guglielmone AA, Gimeno E, Idiart J, Fisher WF, Volpogni MM, Quaino O (1999). Skin lesions and cattle hide damage from *Haematobia irritans* infestations. Med Vet Entomol..

[3] Cupp MS, Cupp EW, Navarre C, Wisnewski N, Brandt KS, Silver GM (2004). Evaluation of a recombinant salivary gland protein (thrombostasin) as a vaccine candidate to disrupt blood-feeding by horn flies. Vaccine..

[4] Fontaine A, Diouf I, Bakkali N, Misse D, Pages F, Fusai T (2011). Implication of haematophagous arthropod salivary proteins in host-vector interactions. Parasit Vectors..

[5] Steen NA, Barker SC, Alewood PF (2006). Proteins in the saliva of the Ixodida (ticks): pharmacological features and biological significance. Toxicon..

[6] Francischetti IM, Sa-Nunes A, Mans BJ, Santos IM, Ribeiro JM (2009). The role of saliva in tick feeding. Front Biosci..

[7] Sá-Nunes A, Oliveira CJF, Kini RM, McLane MA, Clemetson KJ, FSJr M, Morita T (2010). Sialogenins and other immunomodulators derived from blood feeding parasites. Toxins and Hemostasis: From Bench to Bedside.

[8] Wei L, Huang C, Yang H, Li M, Yang J, Qiao X (2015). A potent anti-inflammatory peptide from the salivary glands of horsefly. Parasit Vectors..

[9] Abdeladhim M, Kamhawi S, Valenzuela JG. What’s behind a sand fly bite? The profound effect of sand fly saliva on host hemostasis, inflammation and immunity. Infect Genet Evol. 2014;28:691–703. 10.1016/j.meegid.2014.07.028 PMC4562216 25117872

[10] Cupp EW, Cupp MS, Ribeiro JM, Kunz SE (1998). Blood-feeding strategy of *Haematobia irritans* (Diptera: Muscidae). J Med Entomol..

[11] Zhang D, Cupp MS, Cupp EW (2002). Thrombostasin: purification, molecular cloning and expression of a novel anti-thrombin protein from horn fly saliva. Insect Biochem Mol Biol..

[12] King TP, Spangfort MD (2000). Structure and biology of stinging insect venom allergens. Int Arch Allergy Immunol..

[13] Ameri M, Wang X, Wilkerson MJ, Kanost MR, Broce AB (2008). An immunoglobulin binding protein (antigen 5) of the stable fly (Diptera: Muscidae) salivary gland stimulates bovine immune responses. J Med Entomol..

[14] Wang XY, Ribeiro JMC, Broce AB, Wilkerson MJ, Kanost MR (2009). An insight into the transcriptome and proteome of the salivary gland of the stable fly *Stomoxys calcitrans*. Insect Biochem Molec..

[15] Nestle FO, Di Meglio P, Qin JZ, Nickoloff BJ (2009). Skin immune sentinels in health and disease. Nat Rev Immunol..

[16] Pasparakis M, Haase I, Nestle FO (2014). Mechanisms regulating skin immunity and inflammation. Nat Rev Immunol..

[17] Shapouri-Moghaddam A, Mohammadian S, Vazini H, Taghadosi M, Esmaeili SA, Mardani F (2018). Macrophage plasticity, polarization, and function in health and disease. J Cell Physiol..

[18] Breijo M, Pastro L, Rocha S, Ures X, Alonzo P, Santos M (2016). A natural cattle immune response against horn fly (Diptera: Muscidae) salivary antigens may regulateparasite blood intake. J Econ Entomol..

[19] Edgar RC (2004). MUSCLE: multiple sequence alignment with high accuracy and high throughput. Nucleic Acids Res..

[20] Hall TA (1999). BioEdit: a user-friendly biological sequence alignment editor and analysis program for Windows 95/98/NT. Nucl. Acids. Symp. Ser..

[21] Sa-Nunes A, Medeiros AI, Sorgi CA, Soares EG, Maffei CM, Silva CL (2007). Gr-1+ cells play an essential role in an experimental model of disseminated histoplasmosis. Microbes Infect..

[22] Sá-Nunes A, Bizzarro B, Egydio F, Barros MS, Sesti-Costa R, Soares EM (2016). The dual effect of paradoxical sleep deprivation on murine immune functions. J Neuroimmunol..

[23] Bizzarro B, Barros MS, Maciel C, Gueroni DI, Lino CN, Campopiano J (2013). Effects of *Aedes aegypti* salivary components on dendritic cell and lymphocyte biology. Parasit Vectors..

[24] Sá-Nunes A, Bafica A, Antonelli LR, Choi EY, Francischetti IM, Andersen JF (2009). The immunomodulatory action of sialostatin L on dendritic cells reveals its potential to interfere with autoimmunity. J Immunol..

[25] Forstermann U, Sessa WC (2012). Nitric oxide synthases: regulation and function. Eur Heart J..

[26] MacMicking J, Xie QW, Nathan C (1997). Nitric oxide and macrophage function. Annu Rev Immunol..

[27] Gwakisa P, Yoshihara K, Long To T, Gotoh H, Amano F, Momotani E. Salivary gland extract of *Rhipicephalus appendiculatus* ticks inhibits in vitro transcription and secretion of cytokines and production of nitric oxide by LPS-stimulated JA-4 cells. Vet Parasitol. 2001;99:53–61. 10.1016/s0304-4017(01)00445-9 11445155

[28] Urioste S, Hall LR, Telford SR 3rd, Titus RG. Saliva of the Lyme disease vector, *Ixodes dammini*, blocks cell activation by a nonprostaglandin E2-dependent mechanism. J Exp Med. 1994;180:1077–85. 10.1084/jem.180.3.1077 PMC2191645 8064226

[29] Kopecký J, Kuthejlová M. Suppressive effect of *Ixodes ricinus* salivary gland extract on mechanisms of natural immunity in vitro. Parasite Immunol. 1998;20:169–74. 9618727

[30] Ferreira BR, Silva JS (1998). Saliva of *Rhipicephalus sanguineus* tick impairs T cell proliferation and IFN-gamma-induced macrophage microbicidal activity. Vet Immunol Immunopathol..

[31] Mesquita RD, Carneiro AB, Bafica A, Gazos-Lopes F, Takiya CM, Souto-Padron T (2008). *Trypanosoma cruzi* infection is enhanced by vector saliva through immunosuppressant mechanisms mediated by lysophosphatidylcholine. Infect Immun..

[32] Hall LR, Titus RG (1995). Sand fly vector saliva selectively modulates macrophage functions that inhibit killing of *Leishmania major* and nitric oxide production. J Immunol..

[33] Waitumbi J, Warburg A (1998). *Phlebotomus papatasi* saliva inhibits protein phosphatase activity and nitric oxide production by murine macrophages. Infect Immun..

[34] Katz O, Waitumbi JN, Zer R, Warburg A (2000). Adenosine, AMP, and protein phosphatase activity in sandfly saliva. Am J Trop Med Hyg..

[35] Norsworthy NB, Sun J, Elnaiem D, Lanzaro G, Soong L (2004). Sand fly saliva enhances *Leishmania amazonensis* infection by modulating interleukin-10 production. Infect Immun..

[36] Pushpanjali TAK, Purkait B, Jamal F, Singh MK, Ahmed G (2016). Direct evidence for role of anti-saliva antibodies against salivary gland homogenate of *P. argentipes* in modulation of protective Th1-immune response against *Leishmania donovani*. Cytokine.

[37] Brodie TM, Smith MC, Morris RV, Titus RG (2007). Immunomodulatory effects of the *Lutzomyia longipalpis* salivary gland protein maxadilan on mouse macrophages. Infect Immun..

[38] Schneider BS, Soong L, Coffey LL, Stevenson HL, McGee CE, Higgs S (2010). *Aedes aegypti* saliva alters leukocyte recruitment and cytokine signaling by antigen-presenting cells during West Nile virus infection. PLoS One..

[39] Liu S, Kelvin DJ, Leon AJ, Jin L, Farooqui A (2012). Induction of Fas mediated caspase-8 independent apoptosis in immune cells by *Armigeres subalbatus* saliva. PLoS One..

[40] Bradley JR (2008). TNF-mediated inflammatory disease. J Pathol..

[41] Croxford AL, Kulig P, Becher B (2014). IL-12-and IL-23 in health and disease. Cytokine Growth Factor Rev..

[42] Kobayashi M, Fitz L, Ryan M, Hewick RM, Clark SC, Chan S (1989). Identification and purification of natural killer cell stimulatory factor (NKSF), a cytokine with multiple biologic effects on human lymphocytes. J Exp Med..

[43] Hsieh CS, Macatonia SE, Tripp CS, Wolf SF, O'Garra A, Murphy KM. Development of TH1 CD4+ T cells through IL-12 produced by *Listeria*-induced macrophages. Science. 1993;260:547–9. 10.1126/science.8097338 8097338

[44] Manetti R, Parronchi P, Giudizi MG, Piccinni MP, Maggi E, Trinchieri G (1993). Natural killer cell stimulatory factor (interleukin 12 [IL-12]) induces T helper type 1 (Th1)-specific immune responses and inhibits the development of IL-4-producing Th cells. J Exp Med..

[45] Tripp CS, Wolf SF, Unanue ER (1993). Interleukin 12 and tumor necrosis factor alpha are costimulators of interferon gamma production by natural killer cells in severe combined immunodeficiency mice with listeriosis, and interleukin 10 is a physiologic antagonist. Proc Natl Acad Sci USA..

[46] Gazzinelli RT, Hieny S, Wynn TA, Wolf S, Sher A (1993). Interleukin 12 is required for the T-lymphocyte-independent induction of interferon gamma by an intracellular parasite and induces resistance in T-cell-deficient hosts. Proc Natl Acad Sci USA..

[47] Seder RA, Gazzinelli R, Sher A, Paul WE (1993). Interleukin 12 acts directly on CD4+ T cells to enhance priming for interferon gamma production and diminishes interleukin 4 inhibition of such priming. Proc Natl Acad Sci USA..

[48] Aggarwal S, Ghilardi N, Xie MH, de Sauvage FJ, Gurney AL (2003). Interleukin-23 promotes a distinct CD4 T cell activation state characterized by the production of interleukin-17. J Biol Chem..

[49] Langrish CL, Chen Y, Blumenschein WM, Mattson J, Basham B, Sedgwick JD (2005). IL-23 drives a pathogenic T cell population that induces autoimmune inflammation. J Exp Med..

[50] Kuthejlova M, Kopecky J, Stepanova G, Macela A (2001). Tick salivary gland extract inhibits killing of *Borrelia afzelii* spirochetes by mouse macrophages. Infect Immun..

[51] Kyckova K, Kopecky J (2006). Effect of tick saliva on mechanisms of innate immune response against *Borrelia afzelii*. J Med Entomol..

[52] Brake DK, Perez de Leon AA (2012). Immunoregulation of bovine macrophages by factors in the salivary glands of *Rhipicephalus microplus*. Parasit Vectors..

[53] Chen G, Severo MS, Sohail M, Sakhon OS, Wikel SK, Kotsyfakis M (2012). *Ixodes scapularis* saliva mitigates inflammatory cytokine secretion during *Anaplasma phagocytophilum* stimulation of immune cells. Parasit Vectors..

[54] Prevot PP, Couvreur B, Denis V, Brossard M, Vanhamme L, Godfroid E (2007). Protective immunity against *Ixodes ricinus* induced by a salivary serpin. Vaccine..

[55] Leboulle G, Rochez C, Louahed J, Ruti B, Brossard M, Bollen A (2002). Isolation of *Ixodes ricinus* salivary gland mRNA encoding factors induced during blood feeding. Am J Trop Med Hyg..

[56] Prevot PP, Beschin A, Lins L, Beaufays J, Grosjean A, Bruys L (2009). Exosites mediate the anti-inflammatory effects of a multifunctional serpin from the saliva of the tick *Ixodes ricinus*. FEBS J..

[57] Xu X, Yang H, Ma D, Wu J, Wang Y, Song Y (2008). Toward an understanding of the molecular mechanism for successful blood feeding by coupling proteomics analysis with pharmacological testing of horsefly salivary glands. Mol Cell Proteomics..

[58] Dennis EA, Norris PC (2015). Eicosanoid storm in infection and inflammation. Nat Rev Immunol..

[59] Grewal IS, Flavell RA (1998). CD40 and CD154 in cell-mediated immunity. Annu Rev Immunol..

[60] Suttles J, Stout RD (2009). Macrophage CD40 signaling: a pivotal regulator of disease protection and pathogenesis. Semin Immunol..

[61] Prates DB, Araujo-Santos T, Luz NF, Andrade BB, Franca-Costa J, Afonso L (2011). *Lutzomyia longipalpis* saliva drives apoptosis and enhances parasite burden in neutrophils. J Leukoc Biol..

[62] Heath WR, Carbone FR (2013). The skin-resident and migratory immune system in steady state and memory: innate lymphocytes, dendritic cells and T cells. Nat Immunol..

[63] Castagnolli KC, Ferreira BR, Franzin AM, de Castro MB, Szabo MP (2008). Effect of *Amblyomma cajennense* ticks on the immune response of BALB/c mice and horses. Ann N Y Acad Sci..

[64] Konik P, Slavikova V, Salat J, Reznickova J, Dvoroznakova E, Kopecky J (2006). Anti-tumour necrosis factor-alpha activity in *Ixodes ricinus* saliva. Parasite Immunol..

[65] Gillespie RD, Dolan MC, Piesman J, Titus RG (2001). Identification of an IL-2 binding protein in the saliva of the Lyme disease vector tick, *Ixodes scapularis*. J Immunol..

[66] Breijo M, Rocha S, Ures X, Pastro L, Alonzo P, Fernández C (2017). Evaluation of hematobin as a vaccine candidate to control *Haematobia irritans* (Diptera: Muscidae) loads in cattle. J Econ Entomol..

