# Supplementary figures and images for: Hematobin is a novel immunomodulatory protein from the saliva of the horn fly Haematobia irritans that inhibits the inflammatory response in murine macrophages
Source: Parasit Vectors. 2018 Jul 27;11:435. doi: 10.1186/s13071-018-3017-z (PMC6064106; doi:10.1186/s13071-018-3017-z)

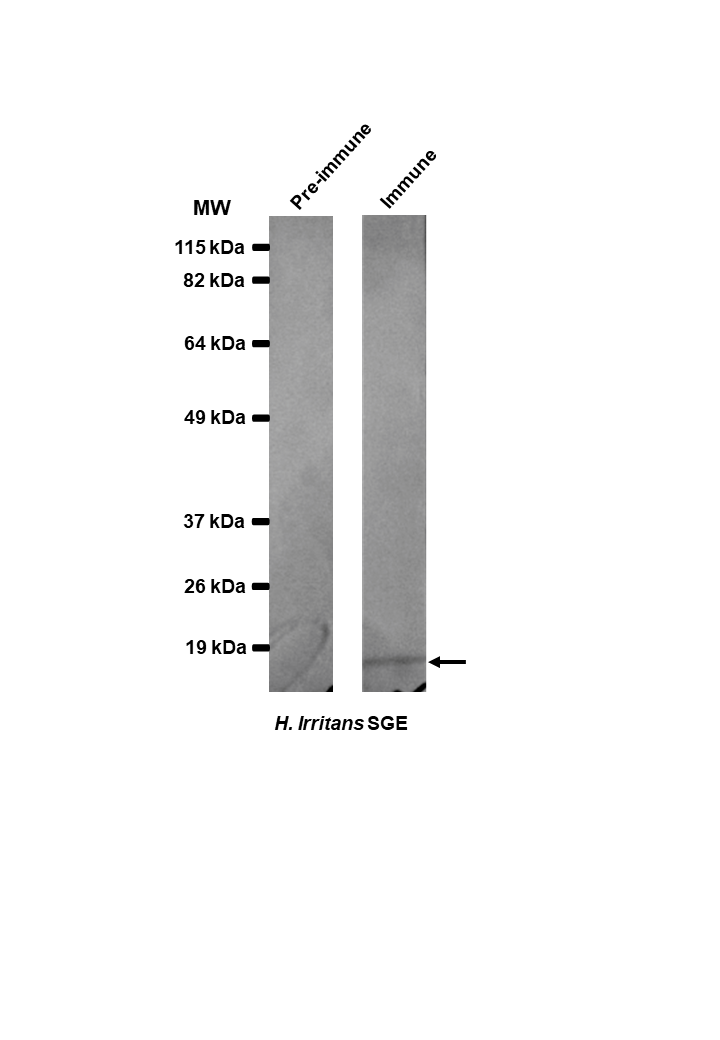

Supplement: Supplementary file 1 — Figure S1. Evaluation of the cross-reactivity between native and recombinant HTB. Whole salivary gland extract of H. irritans was separated on a 12% gradient poly-acrylaminde gel and transferred onto a PVDF membrane. The membranes were blocked with PBS containing 5% of soy milk and were probed with either pre-immune rabbit serum or with serum from rabbits immunized with the recombinant HTB. Then, the membranes were incubated with HRP-conjugated anti-rabbit IgG and bands were detected with 3,3’-Diaminobenzidine. Lane A: pre-immune rabbit serum; Lane B: serum from rabbit immunized with recombinant HTB (immune serum). The immune serum recognized a single band with approximately 15 kDa from the salivary gland extract corresponding to the native protein. MW: molecular weight. (TIF 127 kb) [file 13071_2018_3017_MOESM1_ESM.tif]

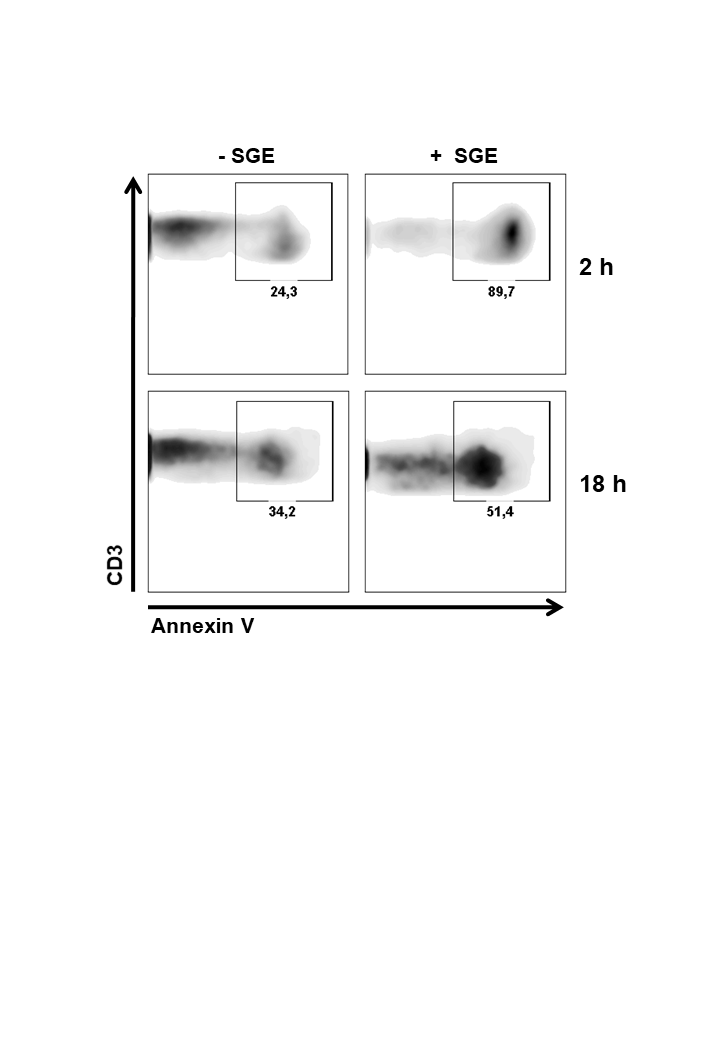

Supplement: Supplementary file 2 — Figure S2. Aedes aegypti SGE induces lymphocyte death in vitro. Spleen cells were cultured in the presence of A. aegypti SGE and stimulated by Con A (0.5 μg/ml, final concentration) according to [23] as a positive control for the assay presented in Fig. 5. Flow cytometry evaluated annexin V+ events in CD3+-gated cells after 2 h and 18 h. The data are expressed as the percentage of annexin V+ events for each condition. (TIF 115 kb) [file 13071_2018_3017_MOESM2_ESM.tif]
